# The International Classification of Vestibular Disorders: Achievements, challenges, and future directions

**DOI:** 10.1177/09574271251313803

**Published:** 2025-01-08

**Authors:** Diego Kaski, Alexander A Tarnutzer, Yuri Agrawal, John Carey, Yoon-Hee Cha, Scott DZ Eggers, Joseph Furman, Hyun Ah Kim, Ji-Soo Kim, Thomas Lempert, Jose A López-Escámez, Mans Magnusson, David E Newman-Toker, Barry M Seemungal, Jeffrey P Staab, Michael Strupp, Raymond van de Berg, Michael von Brevern, Bryan K Ward, Alexandre Bisdorff

**Affiliations:** 1 SENSE Research Unit, Department of Clinical and Movement Neurosciences, Institute of Neurology, University College London, London, UK; 2 Neurology, Cantonal Hospital of Baden, Baden, Switzerland; 3 Faculty of Medicine, University of Zurich, Zurich, Switzerland; 4 Department of Otolaryngology-Head and Neck Surgery, 1466 Johns Hopkins University, School of Medicine, Baltimore, USA; 5 Department of Otorhinolaryngology, 1466 Johns Hopkins University, School of Medicine, Baltimore, USA; 6 Department of Neurology, University of Minnesota, Minneapolis, MN, USA; 7 Department of Neurology, Mayo Clinic, Rochester, MN, USA; 8 Department of Otolaryngology, University of Pittsburgh, Pittsburgh, PA, USA; 9 Department of Neurology, Keimyung University Dongsan Hospital, Daegu, South Korea; 10 Department of Neurology, Seoul National University College of Medicine, Dizziness Center, 26725 Seoul National University Bundang Hospital, Seongnam, South Korea.; 11 Department of Neurology, Schlosspark-Klinik, Berlin, Germany; 12 Meniere Disease Neuroscience Research Program, Faculty of Medicine and Health, School of Medical Sciences, The Kolling Institute, University of Sydney, Sydney, New South Wales, Australia; 13 Division of Otolaryngology, Department of Surgery, Instituto de Investigación Biosanitaria, ibs.GRANADA, Universidad de Granada, Granada, Spain; 14 Sensorineural Pathology Programme, Centro de Investigación Biomédica en Red en Enfermedades Raras, CIBERER, Madrid, Spain; 15 Department of Otorhinolaryngology and Clinical Sciences, Lund University & Skane University Hospital, Lund, Sweden; 16 Rigshospitalet and Danish Technological University, Copenhagen, Denmark; 17 Department of Neurology, 1466 Johns Hopkins University, School of Medicine, Baltimore, MD, USA; 18 Department of Epidemiology, Johns Hopkins Bloomberg School of Public Health, Baltimore, MD, USA; 19 Centre for Vestibular Neurology, Department of Brain Sciences, 4615 Imperial College London, London, UK; 20 Departments of Psychiatry and Psychology and Otorhinolaryngology-Head and Neck Surgery, Mayo Clinic, Rochester, MN, USA; 21 Department of Neurology and German Center for Vertigo and Balance Disorders, LMU Hospital, LMU Munich, Munich, Germany; 22 Department of Otorhinolaryngology and Head and Neck Surgery, Division of Vestibular Disorders, Maastricht University Medical Center, Maastricht, Netherlands; 23 Private Practice of Neurology and Department of Neurology, Charité, Berlin, Germany; 24 Department of Otolaryngology-Head and Neck Surgery, 1466 Johns Hopkins University, School of Medicine, Baltimore, MD, USA; 25 Clinique du Vertige, Centre Hospitalier Emile Mayrisch, Esch-sur-Alzette, Luxembourg

**Keywords:** Bárány Society, International Classification of Vestibular Disorders, dizziness, vertigo, unsteadiness

## Abstract

In 2007, the Bárány Society embarked on a project to establish definitions of vestibular syndromes and disorders based on best available evidence, referred to as the International Classification of Vestibular Disorders (ICVD). Since then, numerous publications providing consensus-driven diagnostic criteria for vestibular symptoms, syndromes, and disorders have been published. Here, we reflect on the rationale for developing the ICVD as well as its subsequent achievements, challenges, and outlook. In this summary of the work of the ICVD to date, the authors will focus on practical aspects to help improve the utility and applicability of these diagnostic criteria moving forward.

## Introduction

Accurate diagnosis of a patient with vertigo, dizziness, or unsteadiness can be challenging, from acute symptoms in emergency departments and primary care, through to chronic symptoms across all settings. One of the challenges is the heterogeneity of symptoms on clinical presentation and the wide range of etiologies underlying these leading symptoms.^1–3^ Patients often struggle to articulate their sensations^4^ but healthcare professionals must interpret the reporting of subjective experiences to narrow the differential diagnosis and determine an underlying pathology, particularly for conditions lacking diagnostic confirmatory tests.

Diagnostic or operational criteria are thus of particular importance where no or non-specific biomarkers exist for a given condition, as they have been successfully employed in functional gastrointestinal disorder classifications,^5^ psychiatry,^6^ and headache disorders.^7^ The vestibular community has long recognized that researchers and clinicians use certain terms inconsistently (e.g., dizziness and vertigo) with different expressions used in different languages. This is one reason why, for instance, inclusion criteria for epidemiological studies or clinical trials for certain conditions, such as vestibular manifestations of migraine, were heterogeneous. In 2007, the Bárány Society thus embarked on a project to establish definitions of vestibular syndromes and disorders based on the best available evidence at that time. The development of these definitions and diagnostic criteria followed a rigorous procedure with an internal review process under the leadership of a dedicated classification committee followed by review by the larger Bárány Society membership.

The diagnosis of vestibular disorders is aided by the use of formalized diagnostic criteria, including symptoms, signs, and available tests for confirmation or exclusion. This process also involves determining levels of diagnostic certainty and applying exclusion criteria in the differential diagnosis. To streamline this approach, the Classification Committee of the Bárány Society (CCBS) was established to create the International Classification of Vestibular Disorders (ICVD).^8^ The CCBS formed various subcommittees to address specific vestibular disorders and related topics. Their goal was to standardize terminology, define key vestibular symptoms, and develop consensus criteria for specific vestibular disorders based on these definitions.

In crafting diagnostic criteria that reflect the range of vestibular disorder presentations, the committee paid close attention to specificity, sensitivity, overlaps between conditions, and the limitations of existing evidence. Each subcommittee was chaired by a Bárány Society member with recognized expertise in the relevant area. The chairperson’s role included forming a writing team consisting of experts from three different continents and two different specialties. After drafting the criteria, members of the Bárány Society were invited to review and provide feedback before the CCBS gave final approval. For some disorders, the Bárány Society collaborated with other professional societies (e.g., to jointly draft criteria for vestibular migraine and to review criteria for persistent postural perceptual dizziness). All diagnostic criteria were intended to be reviewed periodically and updated in light of emerging research. Initially, they were expected to serve as a framework for researchers focusing on clinical vestibular studies, with the anticipation that they would soon influence clinical practice, similar to the evolution of diagnostic criteria in the field of headache disorders.

Here, we self-reflect on the motivation of the initiative, achievements, challenges, and the outlook of the ICVD. Perhaps one panoptic reflection on the ICVD, more than 15 years after its inception, is whether it has been widely accessible and cited. The criteria have been published in the Journal of Vestibular Research, where there have been 4585 citations (SCOPUS), suggesting that ICVD criteria have had high uptake and have responded to a widely felt need. However, no system is perfect, and reflecting on its limitations and challenges is equally important. Moreover, usability may not necessarily translate into improved patient outcomes.

## ICVD structure

**Figure 1.**
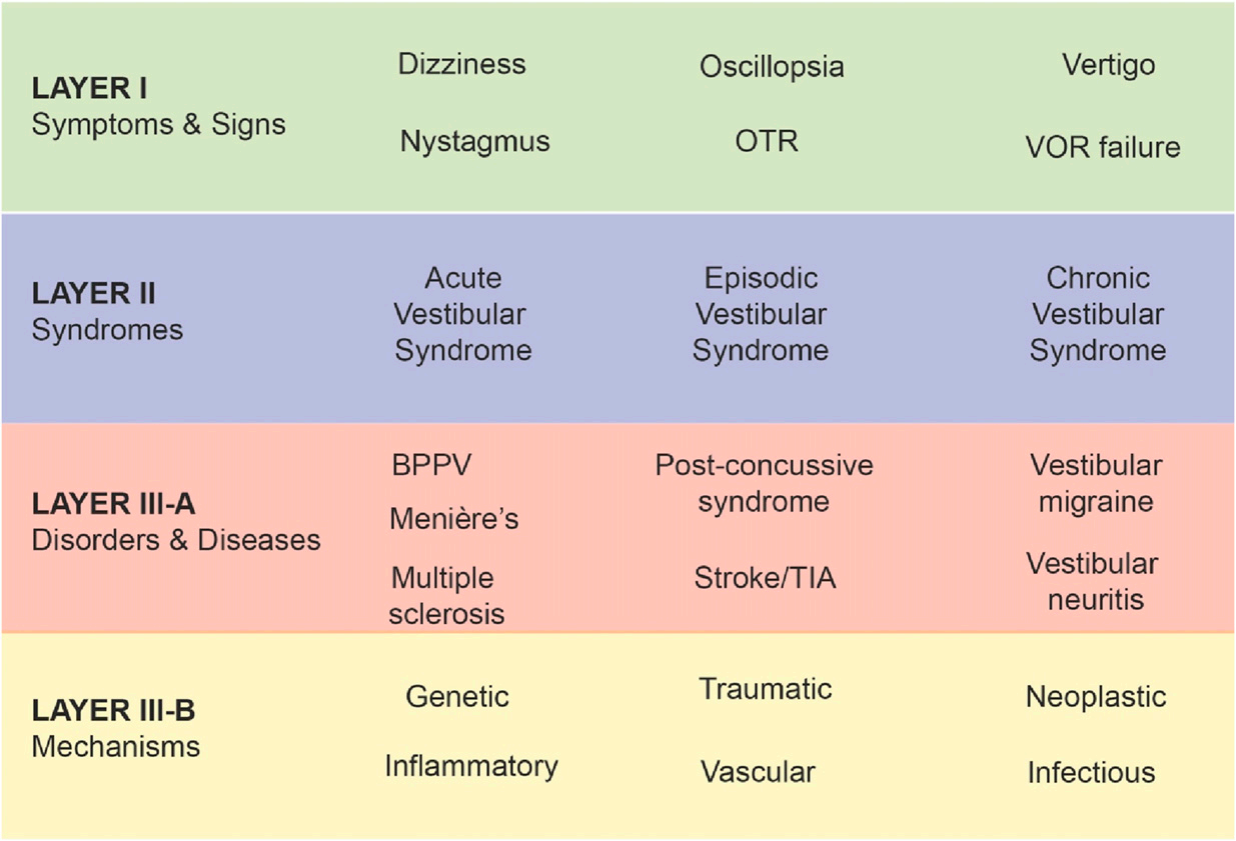
Four-layer framework of the International Classification of Vestibular Disorders. *BPPV:* benign paroxysmal positional vertigo; *OTR:* ocular tilt reaction; *TIA*: transient ischemic attack; *VOR:* vestibular ocular reflex.

An early challenge related to choosing which axes or dimensions on which to classify vestibular disorders, including their possible intersections (e.g., central vs peripheral conditions, with/without accompanying symptoms, duration of symptoms, etc.), The need for structured definitions incorporating criteria across multiple dimensions to inform epidemiologic, diagnostic, and therapeutic research is more obvious for disciplines that rely heavily on symptom-driven syndromic diagnosis, such as psychiatry and headache neurology, where there are currently no confirmatory diagnostic tests available for most defined syndromes. Similarly, for vestibular disorders, a classification scheme along a single dimension (like central vs peripheral site of lesion or presence/absence of accompanying symptoms) would not adequately capture the complexity of the illness manifestations. With these considerations, the ICVD adopted a conceptual model of vestibular disorders as follows (Figure 1):

Layer I—symptoms and signs;

Layer II—clinical syndromes;

Layer III—diseases and disorders;

Layer IV—pathophysiologic mechanisms.

Clinicians and researchers may need to structure their approach by starting with signs and symptoms, while others might prioritize specific diseases or underlying pathophysiological mechanisms, such as genetic or vascular risk factors. Although the primary focus may differ based on the clinical or research context—such as individual case formulation, designing a diagnostic care pathway, or investigating a new mechanism of action-based therapy—the necessity for such an approach remains universal. Such a structure allows the user to approach the classification from any point. This structure also enables new advances in a field to be fed directly into the classification system without requiring major reorganization of the criteria. We reflect next on the classification system by exploring the individual layers.

### Layer I: Symptoms and signs

Layer I of the ICVD includes definitions for specific vestibular symptoms and was the foundation for developing all subsequent definitions (7). Vestibular symptoms do not per se have specific localizing value, as they may arise from dysfunction of the inner ear and its immediate infratentorial connections in the brain or rarely supratentorial brain lesions. Vestibular symptoms can all be caused by a range of general medical (e.g., hypoglycemia, anemia), neurological (e.g., migraine, epilepsy, dysautonomia, cerebellar and extrapyramidal disorders), cardiovascular (e.g., orthostatic hypotension, arrhythmias), respiratory (e.g., obstructive sleep apnea), psychiatric (e.g., panic attacks, generalized anxiety), and other conditions.^3^ Secondary symptoms, such as nausea, fatigue, anxiety, and depression, which frequently accompany vestibular disturbances^9^ were not included in ICVD definitions as they were believed to lack sensitivity and specificity to add diagnostic value. Emerging data indicate that brain disease (and aging) alters patients’ symptom reports of vestibular conditions resulting in dramatic underdiagnosis of common diagnoses such as benign paroxysmal positional vertigo (BPPV), in vulnerable groups.^10–13^

### Layer II: Clinical syndromes

The committee agreed on three cardinal syndromes distinguished by the duration of symptoms to define Layer II. These are (i) acute vestibular syndrome (AVS) comprised of monophasic diseases of acute onset lasting days to weeks, (ii) episodic vestibular syndrome (EVS) encompassing illnesses manifesting recurrent attacks of vestibular symptoms each lasting seconds to days, and (iii) chronic vestibular syndrome (CVS) including conditions in which symptoms persist for a minimum of 3 months. These three duration-based syndromes support clinical reasoning that more quickly and less ambiguously narrows the differential diagnosis than qualitative symptom-based approaches promulgated since the 1950s that attempted to sort diagnoses by distinguishing vertigo from dizziness from unsteadiness^14^ when in reality no vestibular symptom has pathognomonic specificity as patients may describe various combinations of vertigo, dizziness, and unsteadiness, regardless of diagnosis.

While the classification system has been successful in linking the symptoms/signs of Layer I to the syndromes of Layer II based on the duration of vestibular symptoms rather than qualitative distinctions, the definitions of persistent postural perceptual dizziness (PPPD) and mal de débarquement syndrome (MdDS) showed that symptom quality could not be ignored entirely as neither of these disorders includes spinning vertigo. In fact, bouts of spinning vertigo in patients who meet criteria for PPPD or MdDS appear to indicate the presence of co-existing illness. Thus, it will remain important to describe symptom quality carefully in classifications under development and in future revisions of established classifications. Consistent with the plan to periodically update the ICVD, a new subtype of vertigo, haptic vertigo, will be defined and included in the definition of vertigo when it undergoes its scheduled revision. This new subtype reflects a core manifestation of vertigo in MdDS, in which patients report sensations that the supporting surface is undulating or oscillating.

The syndromic layer of the ICVD may be useful for epidemiologic investigations and as a first branch point in diagnostic algorithms, but it is insufficient for complete clinical management.^15^ For example, the AVS encompasses both peripheral and central diseases, the former typically non-life threatening (e.g., acute unilateral peripheral vestibulopathy [AUPV]/vestibular neuritis) whereas the latter may be fatal (e.g., brain stem or cerebellar infarction). Therefore, diagnostic algorithms must include appropriate examinations (e.g., “HINTS” bedside assessment^16^) that can distinguish among diseases and disorders with similar temporal presentations and partially overlapping symptoms and signs. This is particularly true for spontaneous onset AVS and spontaneous onset EVS where there may be more ambiguity in the differential diagnosis than exists for triggered EVS where classic precipitants exist (e.g., changes in head position in patients with BPPV). To complete the integration of syndromic classifications into diagnostic algorithms, it is important to incorporate red flags associated with potentially sinister pathologies, such as associated neurological dysfunction, severe gait ataxia, acute unilateral loss of hearing, and vascular risk factors.^17–19^

### Layer III: Diseases and disorders

The ICVD committee decided that describing all diseases or disorders manifesting vertigo, unsteadiness, dizziness, or balance symptoms would necessarily include illnesses outside the scope of the classification system (i.e., conditions not primarily of vestibular origin), and would require long lists of differential diagnoses that would not be practical or useful to clinicians or researchers. Instead, the ICVD classification committees started with common diseases and disorders in neuro-otology and vestibular medicine (e.g., BPPV, Menière’s disease), expanded to clinically recognized variations of other disorders (e.g., vestibular migraine, vestibular manifestations of stroke), formalized definitions of entities that lacked a unifying criteria (e.g., PPPD), and defined disease states that are debilitating forms of typically transient symptoms (e.g., MdDS, motion sickness disorder). Future efforts will address other medical and surgical disorders that manifest vestibular symptoms, such as traumatic brain injury,^10,20^ which could not be defined until the core group of primary vestibular diseases and disorders was codified. It is expected that the definitions of these diseases and disorders may change with findings from future investigations in an iterative process.

All Layer III definitions were built from symptoms and signs defined in Layer I, and some explicitly incorporated the syndromes defined in Layer II. Some common conditions such as vestibular migraine are defined only by symptoms elicited in patients’ clinical histories because no diagnostic tests currently exist. Others include physiological measures. The definition of Menière’s disease has a criterion for fluctuating low-frequency loss on audiograms. The definition of superior semicircular canal dehiscence includes negative bone conduction thresholds, enhanced vestibular evoked myogenic potentials, or pressure-induced eye movements in the plane of the affected canal. A challenge for episodic disorders (e.g., BPPV) is that physical examinations and vestibular testing often are performed between episodes; although diagnostic criteria made allowances for this situation, future revisions may incorporate signs obtained from portable or wearable devices used by clinicians at the bedside or emergency service or self-recorded by patients in their homes.^21,22^ Examples include portable bedside oculographic and video head impulse testing equipment and wearable goggles or devices to assist with recordings from mobile phones.^23^ Advances in neuroimaging fuel the need to understand the utility and limitations of including imaging findings in diagnostic criteria. Vascular vertigo/dizziness criteria^18^ may need to be updated as new studies emerge, further elucidating the relationship between supratentorial strokes and vertigo without nystagmus^24^ or those linking small vessel disease of the brain to unsteadiness and dizziness.^25,26^ Imaging advances also may yield findings of sufficient sensitivity and specificity to be incorporated into future updates of diagnostic criteria for peripheral disorders, such as Menière’s disease^27^ and AUPV.^28^

### Layer IV: Mechanisms

Layer IV encompasses the pathoanatomic, pathophysiologic, and etiologic mechanisms that underlie vestibular disorders. This layer is the last to be fully developed, given current limitations in understanding the pathophysiology of many vestibular disorders. It is also, however, anticipated to expand the most as scientific discoveries progress. This layer was designed with the expectation that, in the future, clinical phenomena (such as symptoms and signs) could be linked to mechanistic understanding (e.g., mechanical models or computer simulations in BPPV or genetic mutations in ataxia syndromes) for diagnostic and treatment purposes, as recently shown for downbeat nystagmus.^29^ Such advancements might bypass the intermediate steps currently necessary in the diagnostic process (e.g., diagnosing Menière’s disease based on symptoms, signs, and test results without understanding the underlying mechanisms) and lead to the deployment of precision medicine treatments without regard to “syndrome” or “disease.”

### Current challenges in the use of the 4-layer ICVD structure

The process of developing the ICVD also allowed the Bárány Society to identify concepts in the field that require more development. For example, the CCBS recently addressed the controversial condition of cervical dizziness.^30^ After a thorough evaluation, the classification committee opted to publish a position paper that outlined current limitations for defining cervical dizziness because of insufficient evidence to support a unique disease or disorder linking illusory sensations of self-motion with neck pain or signs of primary neck disease.^30^ Rather, the committee recommended that the concept be tested further in dedicated research studies, to include clinical epidemiologic investigations aimed at identifying patients with a unique combination of vestibular and neck-related symptoms and signs that stand apart from other disorders. This approach reflects a view of the ICVD as a pragmatic compendium of the state of knowledge about vestibular diseases and disorders and their relationships with one another that can be judged in part by how each entity is embedded within its 4-layer structure. To be added as a new disease or disorder a proposed condition must encompass a group of patients with manifestations of illness not captured elsewhere, while allowing for the common occurrence of comorbidity.

All existing ICVD definitions of vestibular diseases and disorders include the criterion “not better accounted for by another disorder” (taken from the International Classification of Headache Disorders). Importantly, this criterion does *not* suggest that ICVD diagnoses should be approached by a “rule-out” diagnostic process or are “diagnoses of exclusion” as this criterion is sometimes misinterpreted. Rather, this criterion emphasizes the need to ensure that relevant differential diagnoses are considered in the diagnostic process. All ICVD conditions include a paragraph on what differential diagnosis to consider. Cognizant of the intent to enhance specificity, the practical application of this criterion can be challenging due to subjectivity, the dynamic nature of the field, and the potential for comorbidities. The need to rule out other vestibular disorders before confirming a diagnosis should not promote diagnostic delays, particularly when timely interventions may be crucial for optimal treatment outcomes. How far should healthcare professionals pursue the elimination of other causes where the diagnosis is otherwise clear? Individual preference may dictate this, but so do healthcare structures and legal implications. Striking a balance between specificity and flexibility in diagnostic criteria is essential to accommodate the complexities of vestibular syndromes and ensure accurate diagnoses and timely implementation of needed therapies.

Comorbidity is common in neuro-otology and vestibular medicine and can be encountered in four ways: (i) patients may have two or more active diseases occurring independently (e.g., BPPV and vestibular migraine [VM], both common disorders), (ii) patients may have multiple vestibular disorders as a result of a single aetiology, for example, in traumatic brain injury multiple vestibular disorders is the rule,^31,32^ (iii) patients may develop one disorder as a sequela of another (e.g., PPPD following AUVP), or (iv) patients also may have symptoms that overlap two conditions (e.g., VM and Menière’s disease). Although this last situation could reflect a chance occurrence of two independent illnesses, it also might occur because of incomplete understanding of shared mechanisms. As such, the ICVD has accepted partially overlapping diagnostic criteria when each defined entity captures a unique group of patients, that is, ones that fulfill diagnostic criteria for only one entity even if others have overlapping symptoms or signs. It is expected that the iterative process of updating the ICVD based on future research findings will update definitions to sharpen diagnostic boundaries but also will clarify when overlapping criteria truly reflect pathophysiological processes that produce dual or sequential manifestations of illness.

Shifts in presenting symptoms may require reclassification of a patient’s condition as a patient’s clinical presentation evolves. This concept is not inherently implemented in the diagnostic criteria proposed by the ICVD, except for AUVP,^33^ but reflects current clinical practice and thus could be emphasized more. One salient example is the overlap between VM and PPPD, disorders that may lie on a continuum, with blurring of the diagnostic margins as VM becomes chronic.^34^ There are, furthermore, arguments that support sub-typing of PPPD, a possible need to reconsider the “postural” element of the definition, and a dislike for the phonetic similarity between BPPV and PPPD, that may require updating of the existing criteria. For other, well-established disorders, such as BPPV, the committee may review the appropriateness of linking pathology to prognosis, and more strongly recommend screening for BPPV irrespective of vertigo complaint in certain predisposed groups—for example, among the elderly, especially with a history of falls^35,36^ or among those with traumatic brain injury, where underdiagnosis rates are as high as 10-fold.^11,13^

A major challenge is identifying which disorders should be defined and how to set boundaries and tackle diagnostic overlap (e.g., VM and Menière’s disease). This requires an engaged committee that sits at the forefront of developments in the field and is sufficiently diverse to capture cross-cultural nuances. A focus should be put on disorders that are both clinically important (e.g., MdDS), underdiagnosed (e.g., VM), or often confused with other entities (e.g., cerebellar stroke vs AUPV). There is still much work to be done to complete the list of disorders in need of definition and moving forward with those that have stalled due to the overlap with existing disorders (e.g., traumatic brain injury-related vestibulopathies). There are currently four new subcommittees that address somewhat less specific vestibular entities such as post-concussion vestibular disorders and combined peripheral and central vestibular disorders. Such entities seem more challenging to address given the range of pathologies involved (e.g., head trauma can lead to combined peripheral and central vestibular dysfunction accompanied by other brain injuries and “psychological trauma”), with a range of underlying mechanisms that may result in measurable lesions (e.g., sudden vestibular loss) or may not result in measurable lesions (e.g., vestibular agnosia).

As we unravel the complexities of vestibular disorders, collaboration among healthcare professionals (especially ENT, neurology, physical therapists, vestibular and balance researchers across multiple fields) and patients becomes crucial. A holistic approach that acknowledges the limitations of current diagnostic criteria while embracing new insights will pave the way for improved diagnostic accuracy and enhanced patient care. While the primary purpose of the ICVD diagnostic criteria is to strengthen research, the hope remains that they will also improve the quality of clinical care of vestibular patients.

Many clinicians will use the ICVD diagnostic criteria frequently in their clinical practice but the threshold for meeting a given condition’s criteria is high to increase specificity. While this allows the study of more homogeneous patient cohorts in research, clinicians may need to adjust their thresholds for diagnosis by being aware of the clinical need or context.

## Future directions

The field of vestibular medicine is dynamic, with constant advancements reshaping our understanding of these disorders. Diagnostic criteria may struggle to keep pace with this evolution, including imaging, genetic biomarkers, and immunological biomarkers for some conditions.^37,38^ As new insights emerge, the criteria may become outdated or fail to capture emerging subtypes of vestibular disorders, necessitating periodic updates and revisions open to paradigm shifts and substantial amendments. Equally, including new findings or diagnostic procedures will need to be considered in the context of existing understanding or models of disease. Future consensus guidelines should also consider emerging conditions^39^ and perhaps also definitions for syndromes that do not yet have clear pathophysiological explanations. Currently, the ICVD consensus papers covering a variety of vestibular disorders have not been updated, with one exception (vestibular migraine, where an update was published after ten years). This is a matter of internal procedure to set a certain number of years when an update needs to take place (as is done for clinical guidelines).

In addition to collaborating with individuals and organizations within the vestibular, neurological, physical therapy, and ENT communities, the Bárány Society has sought to establish cooperation and consensus with scientific associations from related fields such as the International Headache Society. There is, however, room to increase the breadth of collaboration with societies in these fields to include representation on some consensus committees and subcommittees, such as vascular vertigo or hemodynamic orthostatic dizziness/vertigo.^40^

## Concluding remarks

Navigating the labyrinth of vestibular disorder diagnoses requires a delicate balance between the benefits and challenges in applying existing diagnostic criteria. While these criteria offer precision in diagnosis, research advancement, and patient empowerment, they also grapple with the heterogeneity of symptoms, subjectivity in patient reporting, overlap with other conditions, and the evolving nature of our understanding of these disorders. The ICVD position papers seem to have been widely adopted but they have not undergone clinical validation so this seems an important task to be conducted by the international community. The Bárány Society may also consider expanding the scope beyond classifications to include committees on dissemination of information to patients, therapies, and vestibular education. The ICVD should evolve over time to ensure it remains the primary reference for defining vestibular symptoms, syndromes, and diseases for researchers and clinicians in the vestibular field and beyond.

## Statements and declarations
